# *In situ* T regulatory cells and Th17 cytokines in paired samples of leprosy type 1 and type 2 reactions

**DOI:** 10.1371/journal.pone.0196853

**Published:** 2018-06-08

**Authors:** Maurício Barcelos Costa, Emerith Mayra. Hungria, Aline Araújo. Freitas, Ana Lúcia O. M. Sousa, Juliano Jampietro, Fernando A. Soares, Mariane M. A. Stefani

**Affiliations:** 1 Tropical Pathology and Public Health Institute, Federal University of Goiás, Goiânia, Brazil; 2 Hospital A.C. Camargo, Centro Internacional de Pesquisa, São Paulo, São Paulo, Brazil; 3 Rede D'Or Hospitals Network, Anatomic Pathology Department, São Paulo, Brazil; 4 General Pathology, Faculty of Dentistry—University of São Paulo, São Paulo, Brazil; Université Paris Descartes, FRANCE

## Abstract

Leprosy is a complex chronic, infectious dermato-neurological disease that affects the skin and peripheral nerves especially during immuno-inflammatory episodes known as type 1/T1R and type 2/T2R reactions. This study investigated the *in situ* expression of CD25^+^Foxp3^+^ T_reg_ cells and TGF-β1, IFN-γ, IL-17 in leprosy T1R and T2R. T_regs_ were evaluated in 114 skin biopsies from 74 leprosy patients: 56 T1R (28-paired reaction-free/reactional biopsies, 28 unpaired T1R), 18 T2R (12 paired reaction-free/reactional biopsies, 6 unpaired T2R). Double CD25^+^Foxp3^+^immunostained T_reg_ cells obtained by automated platform (Ventana BenchMark XT, Roche, Mannheim, Germany) were counted (Nikon Eclipse E400 2mm^2^). Cytokine expression was evaluated by immunostaining in 96 biopsies (48 paired reaction-free/reactional lesions, 24 T1R, 24 T2R) using rabbit polyclonal anti human TGF-β1, IFN-γ, IL-17 antibodies (Santa Cruz Biotechnology CA, USA). T_reg_ cell counts in leprosy reactional lesions were higher compared to reaction-free (p = 0.002). T_reg_ numbers were higher in T1R compared to paired unreactional T1R lesions (p = 0.001). Similar frequency of T_reg_ was seen in paired reactional *versus* unreactional T2R lesions. Higher expression of TGF-β, IFN-γ and IL-17 was seen in T2R lesions compared to T1R and reaction-free lesions. The increase in T_reg_ cells during T1R suggests a suppressive role to control the exacerbated cellular immune response during T1R that can cause tissue and nerve damage. Evidences of upregulated T_reg_ cells in TR1, which usually occurs in patients with Th1-Th17 immunity and the indications of the expression of Th17/IL-17 in T2R, which develops in patients with Th2-T_reg_ profile, suggest plasticity of T_reg_-Th17 cells populations and a potential role for these cell populations in the immunopathogenesis of leprosy reactions.

## Introduction

Leprosy is a complex chronic, infectious dermato-neurological disease that affects the skin and peripheral nerves. Leprosy can lead to significant impairment of nerve function and permanent deformities, which are the hallmark of the disease [1]. Multidrug therapy (MDT), available since the 80’s has reduced prevalence; however, incidence remains stable indicating active transmission chains of its etiologic agent, *Mycobacterium leprae* [2]. Leprosy still represents an important public health problem in many endemic countries as India and Brazil [3]. The disease is characterized by a wide spectrum of clinical, immunologic, microbiologic and histopathologic manifestations which can be classified as tuberculoid (TT), borderline tuberculoid (BT), borderline-borderline (BB), borderline lepromatous (BL) and lepromatous leprosy [4].

For decades the Th1-Th2 paradigm was associated with leprosy manifestations but more recently other CD4^+^ T cell populations as Th17 and T regulatory cells (T_reg_) have been implicated in the immunopathology of leprosy [5]. According to these, paucibacillary patients (PB) that comprise TT and BT forms, present low bacillary load, few localized lesions and develop inflammatory Th1 type and Th17 cell-mediated immunity (CMI) to *M*. *leprae* with low antibody production [6–8]. On the other end of the spectrum, multibacillary patients (MB), which include BB, BL and LL forms, show multiple skin lesions, high bacillary load, Th2 type immunity with vigorous antibody production and higher expression of T_reg_ cells [9–12].

One of the major complications in the clinical management of leprosy patients is the development of acute immune inflammatory episodes, known as leprosy reactions, during the chronic course of the disease either before, during or after the specific treatment. Type 1 and type 2 reactions (T1R and T2R) are the main manifestations of leprosy reactions. These episodes are responsible for irreversible nerve damage representing the major cause of permanent physical disabilities and deformities [13]. Contradictory results and interpretations have been reported about the role of T_reg_ cells in the immunopathogenesis of leprosy reactions [14–20]. Several relevant methodological differences and more importantly, the use of different compositions of comparative control groups limit comparisons among these studies. Intra-individual comparisons in paired samples, collected in the course of a reactional episode and when the patient is reaction free, provide the best comparative control possible. In this context, the current study describes *in situ* expression of T_reg_ cells and cytokines with emphasis in paired skin biopsies of T1R and T2R leprosy patients.

## Materials and methods

Our study group comprised 74 leprosy patients that developed leprosy reaction including 56 cases of T1R and 18 cases of T2R. Leprosy patients were recruited at a public health reference center (Centro de Referência em Diagnóstico e Terapêutica, CRDT, Goiânia, Goiás State, central West Brazil). All patients were submitted to a complete dermato-neurologic examination by one dermatologist with vast expertise in leprosy diagnosis (ALOMS) and skin biopsies were taken from typical and active leprosy skin lesions. Leprosy patients were classified according to a modified Ridley & Jopling criteria taking into account histopathology, bacilloscopy in the lesion and clinical data.

Case definition for T1R was acute inflammation of pre-existing cutaneous lesions with or without the appearance of new lesions. T1R patients were diagnosed based on clinical signs and symptoms which were not always associated with a specific histopathological finding, so that often the histopathology of T1R lesion was similar to the histopathology of a skin lesion taken from a reaction-free patient of a similar Ridley Jopling category. When histopathological modifications were seen in T1R, these included edema, looser granulomas and sometimes necrosis foci occasionally with bacilli. Case definition for T2R was based on the acute appearance of erythematous nodular skin lesions, tender to touch or painful, in the absence of external stimuli, accompanied by fever with or without peripheral nerve pain and dysfunction. In T2R, the prevailing histopathological findings were the ones characteristic of erythema nodosum leprosum/ENL, such as panniculitis with leukocytoclastic vasculitis in small vessels and neutrophilic infiltrates occasionally with fragmented bacilli, in smaller numbers than found in reaction-free patients.

Based on dermato-neurologic examination, reaction-free patients were defined as the ones who did not have at the moment of examination any sign or symptom of type 1 nor type 2 reactional episodes.

Leprosy patients received standard treatment according to the WHO recommended regimen: 6 doses MDT (rifampicin, dapsone) for paucibacillary patients (< = five skin lesions) to be taken up to 9 months and for multibacillary patients (> = 6 skin lesions) 12 doses MDT (rifampicin, dapsone, clofazimine) during up to 18 months. For type 1 reaction the anti- reaction treatment consisted of standard- 12 weeks course of oral prednisolone (up to 1mg/kg/day). After this period, the dose was gradually reduced, decreasing 10mg each 15 days, up to 5mg/kg which was maintained for 12 weeks. To treat T2R, if not contraindicated (4 women of childbearing age), thalidomide (100-400mg/day) was used at night. Another alternative was the same oral prednisolone treatment used to treat T1R. If T2R occurred with neuritis, thalidomide and prednisolone were used concomitantly.

In our study among 28 T1R patients with paired samples, 20 were diagnosed during the reactional episode when the first skin biopsies were taken. For these patients, the second skin biopsy was taken after a median time of 12 months. The remaining 8 patients that were reaction-free at diagnosis had their first skin biopsy collected at diagnosis and they developed T1R around 9 months after the diagnosis (6–14 months range), when the second skin biopsy was taken. Among the 12 patients that developed T2R, 7 were diagnosed during the reactional episode when the first biopsy was collected and the second biopsy was taken at a mean time of 12 months after the diagnosis/reaction. The remaining 5 patients were reaction free at diagnosis when the first skin biopsy was taken; their T2R occurred at a mean time of 12 months after the diagnosis, when the second biopsy was collected. Leprosy patients were monitored for 18 months after diagnosis and our study did not include extended follow-up. Therefore, during the study period, all patients presented only a single reactional episode and the investigation of possible occurrence of multiple reactional episodes overtime was not part of the study.

The main demographic and clinical features of our study population is depicted in Table 1. Most leprosy patients was male (66.2%, 49 out of 74) and the median age was 48 years (20–75 years range). Patients that developed T1R (n = 56) presented a wide range of clinical manifestations at diagnosis (3 TT, 28 BT, 02 BB, 22 BL and 01 LL) with either negative or low bacillary counts in the lesions. T2R group (n = 18) included 09 BL and 09 LL patients all of them with detectable bacilli in the lesion (median = 3.5, range 1–6).

**Table 1:** Main demographic and clinical features of leprosy patients.

Study groups (total)	Gender(M/F)	Age Median (years, range)	Ridley & Jopling	Bacillary counts/lesion (median, range)
T1R (n = 56)	35/21	49 (20–75)	TT = 03; BT = 28; BB = 02; BL = 22 LL = 01	1 (0–2)
T2R (n = 18)	14/4	42 (20–71)	BL = 09, LL = 9	4 (1–6)

M: male; F: female. MB: multibacillary leprosy; PB: paucibacillary leprosy; T1R: type 1 reaction; T2R: type 2 reaction; Bacillary counts were performed in the biopsy of the skin lesion after Fite-Faraco staining.

### Skin biopsies, histopathology

Biopsies were taken from the edges of well-characterized and infiltrated skin lesions using standard dermatologic biopsy punches (4mm). Fixation was performed with 10% neutral buffered formalin in 4% formaldehyde for 24 hours. Skin biopsy lesions (3–4 μm sections) were stained by hematoxylin-eosin (HE) and by Fite-Faraco for histopathology examination and bacilli detection/counts. The whole inflammatory infiltrate was analyzed and after this, 8 fields of 400X were selected to perform cell/cytokine countings and these 8 fields correspond to 2 mm^2^. In this study, the emphasis of infiltrates of T1R versus T2R was given on Tregs and the selected cytokines.

### Double immunohistochemical detection of CD25^+^Foxp3^+^T_reg_ cells

For the immunohistochemical study, skin biopsies were deparaffinized (*EZPrep*, Roche), treated with *Cell Conditioner* (Roche) and submitted to heat induced antigen retrieval. Double immunostaining reaction to detect CD25/Foxp3 coexpression was performed using an automated platform (Ventana BenchMark XT, Roche, Mannheim, Germany). The ultra-View Universal DAB detection kit (Roche, Germany) was used for CD25 staining with primary antibody–horseradish peroxidase/HRP labeled complex (anti-human monoclonal antibody, clone 4C9, Ventana, Roche, Tucson, AZ, USA) resulting in a brown/black target signal. Foxp3 staining employed anti-Foxp3^+^ antibodies (mice anti-human monoclonal antibodies, clone 236A/E7, Abcam, Cambridge, UK) and the ultra-View Universal Alkaline Phosphatase Red Detection Kit (Roche, Manhein, Germany). The primary antibody–alkaline phosphatase labeled complex was visualized using Fast Red/Naphthol resulting in a bright red target signal. Slides were washed and Hematoxylin II (Roche, Mannheim, Germany) was added, incubated, washed and the Bluing Reagent (Roche, Mannheim, Germany) was added and washed. Slides were then processed by the automated Tissue-Tek® Prisma®/Film® (Sakura, AJ Alphen aan den Rijn, Netherlands).

Quantification of double immunostained CD25^+^Foxp3^+^T_reg_ cells was performed using Nikon Eclipse E400 microscope (8 fields in 400X corresponding to 2mm^2^; values were then divided by 2 to represent counts in 1mm^2^). All quantifications of T_reg_ positive cells were performed by one observer with vast expertise in dermatopathology (MBC), blinded to the patients’ group. Immunostained cells were quantified in areas with and without inflammatory infiltrates and double stained T_reg_ cells were visualized as brown/golden cytoplasm stained by anti CD25 revealed with DAB and violet/magenta nuclei stained Foxp3 revealed by alkaline phosphatase.

### Immunohistochemistry to cytokines detection

Immunostaining of cytokines employed rabbit polyclonal antibodies to human TGF-β1, IFN-γ, IL-17 and IL-10 (Santa Cruz Biotechnology CA, USA). In brief, after deparaffinization, rehydration and blockade of endogenous peroxidase activity and antigen-retrieval, 3-4um sections of skin biopsies were incubated individually for 1 h with rabbit polyclonal antibodies to human TGF-β, IFN-γ, IL-17 and IL-10. Then, the primary antibody–HRP labeled complex (anti-human monoclonal antibody, clone 4C9, Ventana, Roche, Tucson, AZ, USA) was incubated (30 minutes). Color was developed using diaminobenzidine (DAB, Invitrogen Cat. N° 00–2014) chromogen system (5 minutes). Slides were washed and hematoxylin (Roche, Mannheim, Germany) was added, incubated and washed. For each cytokine, positive and negative controls were performed and the numbers of positive cells were counted by examining 1000 cells from multiple fields using Nikon Eclipse E400 microscope (200X). One observer with vast expertise in dermatopathology (MBC) performed all quantifications of cell associated-cytokines blinded to the participants’ clinical status.

### Statistical analyses

Exploratory data analysis, including dot plot, box plot, means and medians were used to analyze the quantifications of T_reg_ cells/mm^2^ and cytokine positive cells among different study groups. We have done a step by step comparison, first analyzing all reactional lesions together versus all reaction-free lesions. From the differences seen in these analyzes, we have conducted further comparisons which were based on stratified groups of patients: T1R versus reaction-free, and T2R versus reaction-free ones and then the T1R, T2R paired analyzes. The fold increase was based on the median values of Treg cells in each group and it was calculated by dividing the higher median number of Tregs by the lower median number of Tregs in each specific case. Statistical significance was assessed by Kruskall-Wallis one way analysis of variance for comparison of multiple groups and Mann-Whitney for comparison between two groups. Results were considered statistically significant when *p*-values <0.05 were obtained.

### Ethical issues

This study was approved by the regional and the national review boards (“Comitê de Ética em Pesquisa Humana e Animal do Hospital das Clínicas da Universidade Federal de Goiás”, protocol# 119/2005 and “Comissão Nacional de Ética e Pesquisa/CONEP/Brasil”, protocols # 4862, 12962). All patients signed an informed consent form before joining the study.

## Results

### CD25^+^Foxp3^+^T_reg_ cell numbers in T1R, T2R compared to reaction-free lesions

**Fig 1:**
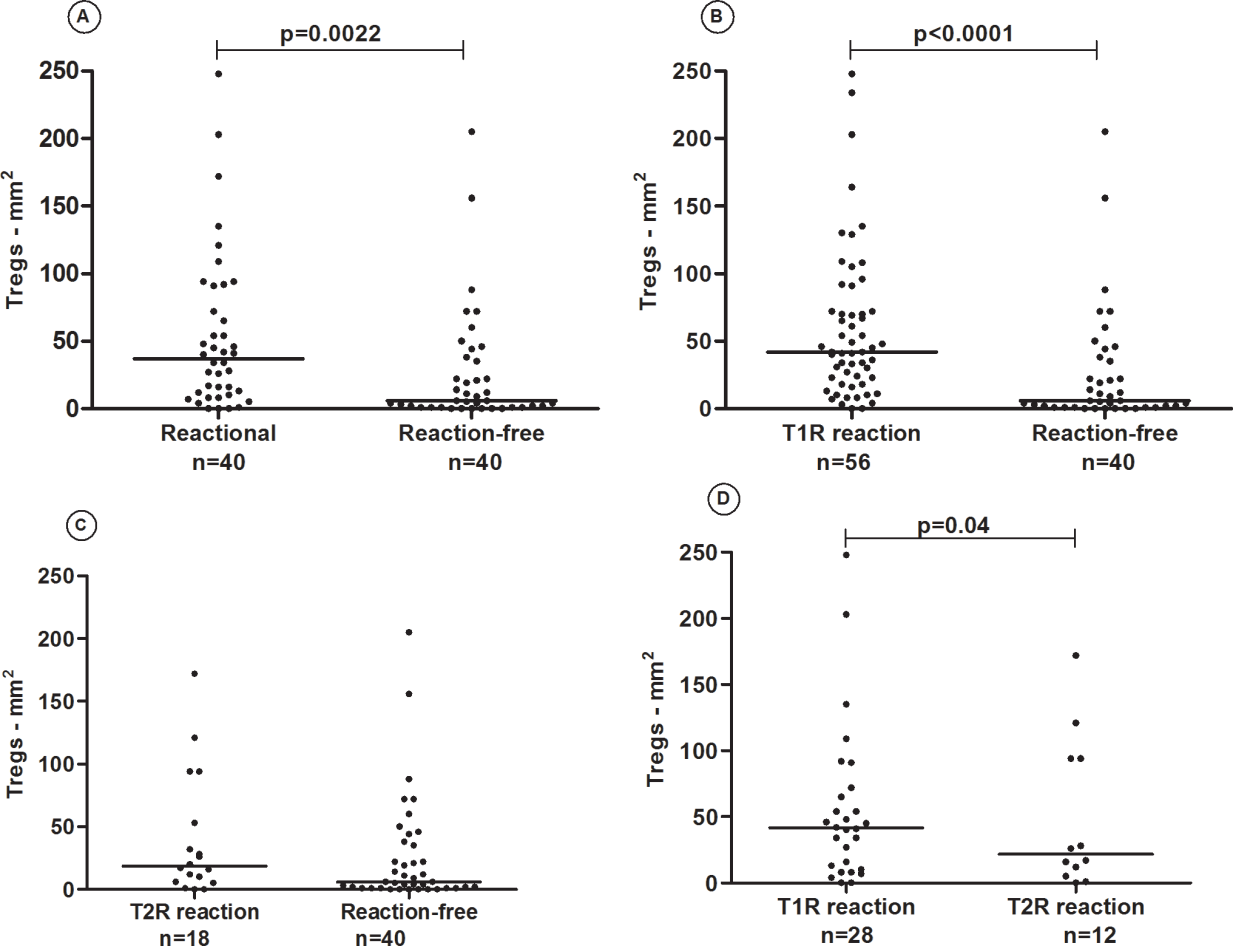
**(**A) CD25^+^ Foxp3^+^T_reg_ cell counts in skin biopsies of T1R+T2R (n = 40) and reaction-free leprosy patients (n = 40). (B) CD25^+^ Foxp3^+^T_reg_ cell counts in biopsies of 56 T1R and 40 unpaired reaction-free patients. (C) CD25^+^ Foxp3^+^T_reg_ cell counts in biopsies of 18 T2R and 40 unpaired reaction-free patients. (D) CD25^+^ Foxp3^+^T_reg_ cells in biopsies of 28 T1R and 12 T2R. The horizontal lines represent the median CD25^+^ Foxp3^+^T_reg_ cell counts.

Higher T_reg_ cell numbers were seen in reactional (28 T1R+12 TR2; n = 40) *versus* the same 40 reaction-free patients (p = 0.002) (Fig 1A). A higher number of T_reg_ cells was observed in 56 T1R patients (28 had paired reactional and reaction-free samples and 28 had an individual reactional biopsy) compared to 40 reaction-free patients (28 reaction-free samples of patients that had T1R + 12 reaction-free samples of patients that had T2R) (p<0.0001) (Fig 1B). However, no difference in T_reg_ cell counts was observed comparing 18 patients with T2R and 40 patients from the reaction-free group (28 reaction-free samples of patients that had T1R + 12 reaction-free samples of patients that had T2R) (Fig 1C). Comparison between T1R (n = 28) and T2R (n = 12) showed higher T_reg_ cell counts in T1R lesions (p = 0.04) (Fig 1D). A two-fold increase in T_reg_ numbers was seen in T1R compared to T2R (T1R: 42cells/mm^2^, T2R: 18.5cells/mm^2^).

**Fig 2:**
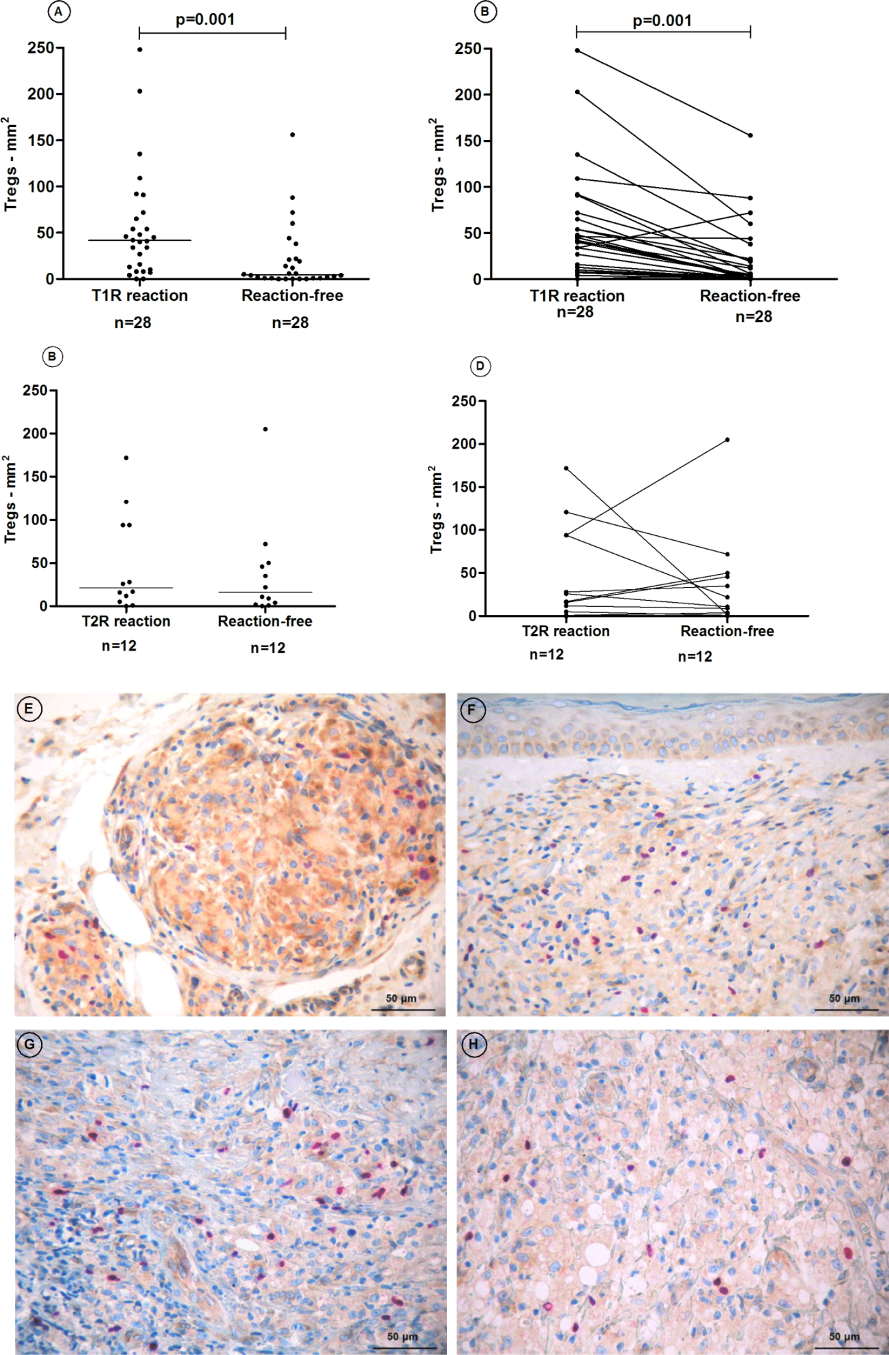
(A-B): CD25^+^ Foxp3^+^T_reg_ cell counts in paired biopsies of T1R and reaction-free patients. The horizontal lines represent the median CD25^+^ Foxp3^+^T_reg_ cell counts. (C-D): CD25^+^ Foxp3^+^T_reg_ cell counts in paired biopsies of T2R and reaction-free patients. (E-F): CD25^+^ Foxp3^+^T_reg_ cells in paired biopsies of a reaction-free BT lesion and during T1R; (G-H): CD25^+^ Foxp3^+^T_reg_ cells in paired biopsies of a reaction-free BL patient and during a T2R. Marks: 50μm.

Intra-individual comparisons in paired reactional and reaction-free biopsies showed higher numbers of T_reg_ in lesions during T1R episodes (p = 0.001) (Fig 2A and 2B). A nine-fold increase of T_reg_ cells was seen during T1R (reaction-free: 4.5cells/mm^2^ *versus* RT1: 41.5cells/mm^2^). In T2R and paired reaction-free lesions intra-individual comparisons showed no difference on T_reg_ cell numbers (reaction-free: 16.5cells/mm^2^ *versus* T2R lesions: 21.5cells/mm^2^, p>0.05) (Fig 2C and 2D). Fig 2 depicts the immunostaining for CD25^+^ Foxp3^+^T_reg_ cells in paired samples of a reaction-free BT patient (Fig 2E) and during T1R (Fig 2F); CD25^+^ Foxp3^+^T_reg_ cells in paired samples of a BL reaction-free patient (Fig 2G) and during T2R (Fig 2H).

Increased numbers of T_regs_ cells in T1R were seen regardless of the moment when the reaction occurred either at diagnosis or during MDT. In T2R, similar T_reg_ counts were observed regardless of the timing of development of reactions, at diagnosis or during MDT (data not show).

### *In situ* expression of TGF-β, IL-17 and IFN-γ in T1R, T2R and reaction-free leprosy lesions

We have investigated the *in situ* expression of TGF-β, IL-17, IL-10 and IFN-γ in paired T1R/ reaction-free lesions (n = 12) and paired T2R (n = 12)/reaction-free lesions. Despite proper standardization of IL-10 staining in positive control samples from human intestine which were run in parallel, no IL-10 staining was seen in leprosy T1R or T2R or reaction-free lesions, while adequate staining for TGF-β, IL-17 and IFN-γ was achieved in leprosy lesions.

**Fig 3:**
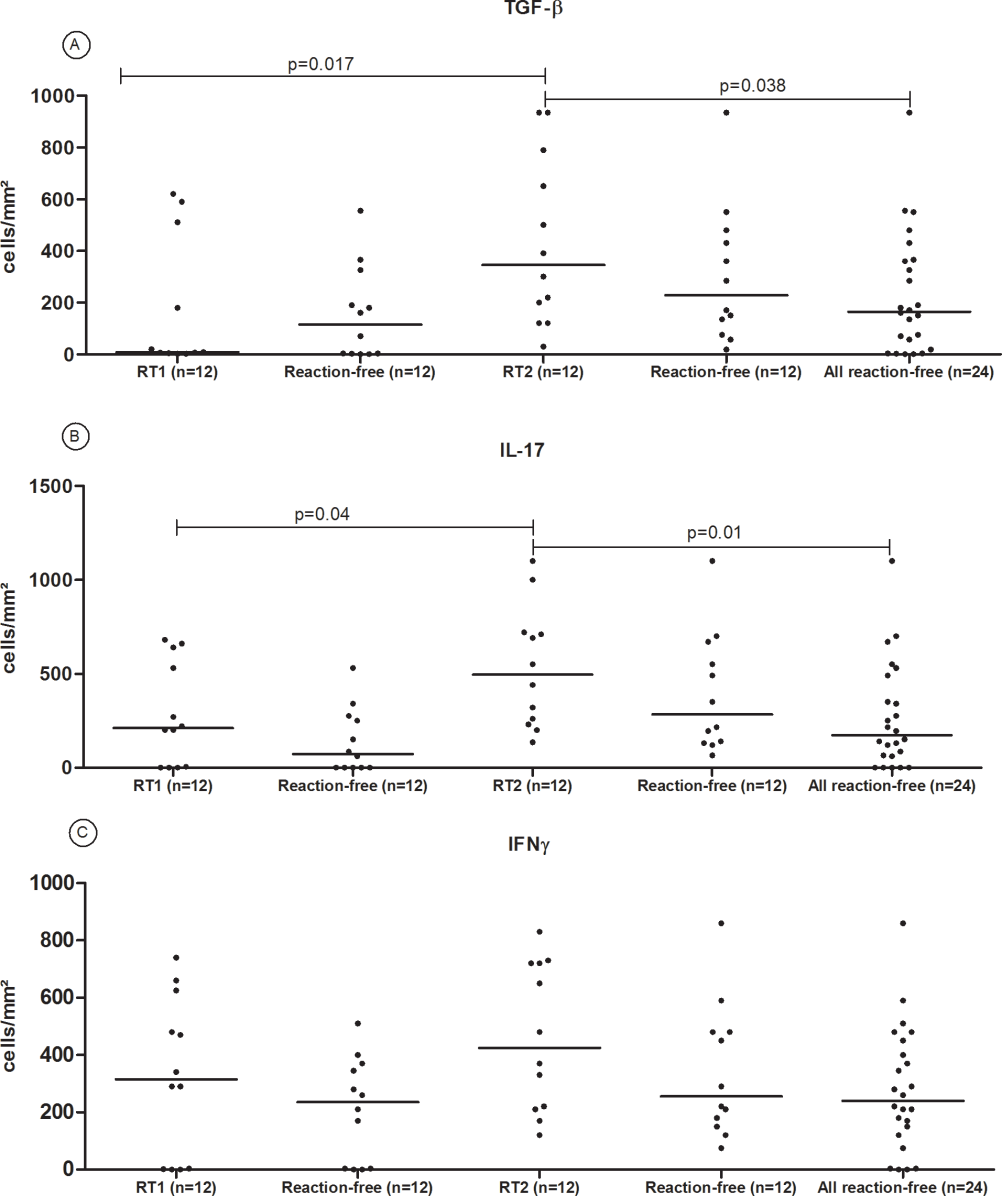
Comparison of the expression of TGF-β (A) IL-17 (B) and IFN-γ (C) in paired skin biopsies of reactional (T1R and T2R) and reaction-free lesions. The horizontal lines represent the median counts of TGF-βL-17 and IFN-γ positive cells.

**Fig 4:**
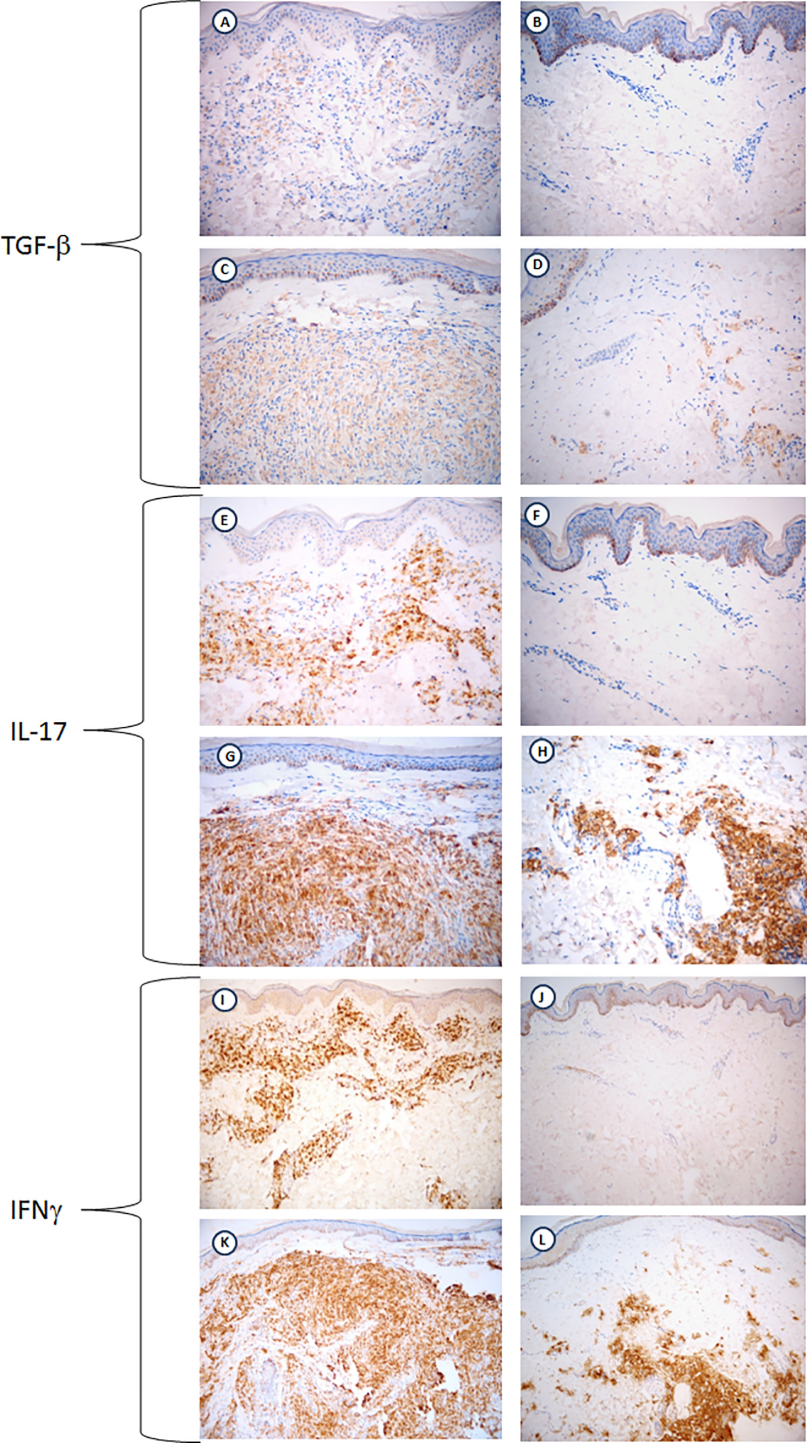
Immunostaining for TGF-βIL-17 and IFN-γin paired skin biopsies of reactional (T1R and T2R) and reaction-free lesions. (A-B): TGF-βin paired biopsies of a patient during T1R and when the patient was reaction-free. (C-D): TGF-βin paired skin biopsies of a patient during T2R and when the patient was reaction-free. (E-F): IL-17 in paired skin biopsies of a patient with T1R and when the patient was reaction-free. (G-H): IL-17 in paired skin biopsies of a patient with T2R and when the patient was reaction-free. (I-J): IFN-γ in paired skin biopsies of a patient with T1R and when the patient was reaction-free. (K-L): IFN-γ in paired skin biopsies of a patient with T2R and when the patient was reaction-free.

The TGF-β expression was increased in T2R lesions compared to RT1 (p = 0.017) and compared to all reaction-free lesions (p = 0.038) (Fig 3A). Similarly, IL-17 expression was higher in T2R lesions compared to T1R (p = 0.04). Statistically significant difference in IL-17 expression was observed comparing T2R lesions to all reaction-free lesions (p = 0.01) (Fig 3B). However, no significant intra-individual difference in IL-17 or TGF-β expression was observed comparing paired T1R-reaction free or T2R-reaction-free samples. So, while higher expression of IL-17 and TGF-β and was seen in T2R compared to T1R lesion, the expression of IFN-γ was similar in T1R, T2R and reaction-free lesions (Fig 3C). *In situ* immunostaining for TGF-β, IL-17 and IFN-γ in paired skin biopsies of T1R-reaction free and T2R-reaction-free lesions is shown in Fig 4.

## Discussion

Our study evaluated the *in situ* expression of both double positive CD25^+^ Foxp3^+^ T_reg_ cells and cytokines in leprosy T1R and T2R with special emphasis on the frequency of T_reg_ cells in paired biopsies collected in the absence of reaction and during the course of a reactional episode. The choice of the control group is a crucial component of comparative studies and we consider that intra-individual comparisons in reactional and reactional-free lesions are probably the best approach to investigate the impact of leprosy reactions upon T_reg_ cell expression. In this study, paired sample analyses have shown an increased expression of T_reg_ during T1R. On the other side, no impact on the lesional expression of T_reg_ cells was seen in T2R.

A previous study showed similar results of increased frequency of T_reg_ cells on five paired samples of T1R but in eight paired samples of T2R, results have shown decreased T_reg_ cell numbers in TR2 [19] contrasting with our results of similar frequencies of T_reg_ with or without T2R. Our results of paired analysis together with other studies indicate that the *in situ* expression of T_reg_ during T2R is either unchanged or decreased. Overall these findings of unchanged or decreased T_reg_ during T2R are compatible with the extensive and somehow uncontrolled clinical manifestations associated with T2R. Under the circumstances of decreased/stable T_reg_ cells expression wider tissue and nerve damage might occur. On the other side, our results of increased T_reg_ numbers during T1R suggest a possible suppressive T_reg_ role to control the exacerbated cell mediated immunity seen in T1R with beneficial rather than detrimental consequences to the host suggesting the possibility of modulating T_reg_ for T1R therapy. However, the challenge remains to translate these laboratory findings of T cell populations and cytokines into the identification of clinically applicable immuno-markers associated with the clinical outcome of leprosy reactions.

Our results have shown increased expression of IL-17 and TGF-β in lesions with T2R compared to T1R and reaction-free lesions. TGF-β along with other pro-inflammatory cytokines (such as IL-6, IL-21 and IL-1β) are known to drive naïve T cells towards Th17 differentiation [21]. Our findings suggest that in T2R, TGF-β may be implicated in the differentiation of Th17 cells however, this hypothesis needs to be confirmed by the investigation of the expression of IL-1β and IL-6. A recent study suggested that IL-12 and IL-23 modulate the plasticity of FoxP3^+^ T_reg_ cells in leprosy, converting T_reg_ cells into Th1 and Th17 [22]. Another report on plasticity of T cells showed that the equilibrium between Th17 and T_reg_ cells in leprosy reactions is disturbed with an increase in Th17 cells which leads to the inflammation and immunopathology associated with T2R [16]. In our study, besides the stable numbers of T_reg_ cells in T2R, the cytokine data indicate that T2R is associated with IL-17, which is a surrogate marker of Th17 activation. Altogether our results of T_reg_ and cytokines expression during leprosy reactions are suggestive of Th17-T_reg_ plasticity however, further experimental studies are required to confirm our hypothesis. T1R and T2R are recognized by extensive differences in the clinical presentation and in the heir histopathological profiles and the role of Treg and Th17 during these episodes is only elusive. Although T1R can occur in any leprosy form, it is more frequent in patients with robust Th1-Th17 immunity, but studies have shown that during T1R, their lesional immune profiles change to Th1-T_reg_ cells. On the other side, T2R occur mainly in patients expressing Th2-T_reg_ immunity, but there are evidences that during T2R their lesional immune profiles convert to Th1-Th17 cells. Therefore, our results corroborate the hypothesis that T2R occurs in a context of stable or decreased T_reg_ and increased Th17 or conversion of T_reg_ into Th17. Nevertheless, so far it is not possible to figure out to which extent the differential expression of Th17 and T_reg_ during TR1 or T2R might be associated with the clinical outcome associated with reactions including nerve and tissue damage, independently of the anti-reaction treatment.

In general, studies evaluating circulating T_reg_ cells suggest an increase of T_reg_ cells during T1R episodes and a decrease in T2R patients, although contradictory results have also been described. These studies may not be comparable for numerous reasons. Different laboratory methodologies and distinct cell markers have been used to identify T_reg_ cell phenotype (CD3, CD4, CD8, CD25, Foxp3, CD127, co-stimulatory molecules). Flow cytometry for surface and or intracellular staining has been used with different gating and acquisition strategies [15, 16, 19, 23]. Also, immunostaining for different T_reg_ cell markers and PCR to investigate the expression of different genes have also been described. T_reg_ expression in leprosy reactions has been described in distinct body compartments either *in situ* T_reg_ in biopsies of skin lesions or circulating systemic T_reg_ in unstimulated fresh and in fresh or thawed stimulated cells [14, 16–18, 24]. Additionally, different antigen preparations have been used for culture stimulation (ML sonicate, PHA) [16, 20]. More importantly, the different compositions of control groups used in the studies clearly impact comparisons. Leprosy reactions have been compared to non-reactional leprosy with similar clinical forms, non-reactional leprosy with all clinical forms, exposed contacts or even healthy individuals. Study groups have included different sample sizes, naïve and MDT treated patients and T2R treated with prednisolone [14, 16, 17, 19, 20, 23]. Although each methodological approach and study design may have advantages and disadvantages, it’s possible that intra-individual paired reaction-free/reactional lesions, as shown in this study, illustrate better the impact of leprosy reaction upon the expression of different T cell populations and cytokines in skin lesions.

One of the first studies about T_reg_ and leprosy reactions indicated a reduced frequency of T_reg_ cells in patients with T2R (n = 6) compared to non-reactional disease forms (BT, TT, BB, BL, LL) [15]. Another study in 43 untreated leprosy patients (TT, BB, LL, T1R, T2R and pure neuritic form) and 40 healthy subjects (controls group) using flow cytometry showed higher expression of circulating T_reg_ cells in patients with T1R [23]. A decrease in T_reg_ cells in T1R and T2R was shown by flow cytometry on PBMCs stimulated with sonicated *M*. *leprae* when compared to controls (non-reactional leprosy) [16]. However, Foxp3 gene expression by quantitative PCR was increased in stimulated PBMC from T1R and T2Rcompared to patients with stable disease. A recent case-control study using flow cytometry and PBMCs in patients with T2R (n = 46) compared to matched non-reactional LL patient controls (n = 31) showed lower rate of CD4+ T_reg_ cells before prednisolone treatment [20]. A retrospective immunostaining study on 20 leprosy cases (TT, BT, BL, LL, BB-T1R, BT-T1R and T2R) showed higher expression of T_reg_ cells in patients affected by T1R (n = 3) compared with patients affected by T2R (n = 3) and reaction-free patients [14]. Immunostaining in 96 leprosy lesions of different forms (Indeterminate/I, TT, BT, BB, BL, LL), T1R (n = 8) and T2R (n = 2) also found an increment of Foxp3 expression in T1R compared to unreactional lesions (I, BT and LL forms) [17]. Recently, the expression of cytokines related to T_reg_ cell profile was evaluated by quantitative PCR in 87 skin biopsies from leprosy patients and showed decreased T_reg_ cells markers in T1R and increased IL-17F, CCL20 and IL-8 in T2R when compared to the respective non-reactional leprosy patients [18].These apparently conflicting results are difficult to be compared may reflect the different methodological approaches and comparison groups used.

In conclusion, our study in paired skin biopsies before and during reaction showed increased numbers of T_reg_ cells during T1R suggesting its beneficial role in the control of exacerbated inflammation and cellular immunity and consequent pathology seen in T1R. On the other hand, we showed stable T_reg_ and an increase of IL-17 pro-inflammatory cytokine in T2R leprosy lesions suggesting the involvement of Th17 cells. Our results suggest a possible plasticity of T_reg_-Th17 populations in leprosy reactions. In human infectious diseases T_reg_ cells may function like as a “double-edged sword” in which both the host and the pathogen may benefit from their function. The pathogen advantage would be because T_reg_ reduces effector immunity leading to disease chronicity. The host advantage can be due to suppressive T_reg_ activity limiting tissue damage caused by inflammation and tissue/nerve damage that occur in leprosy reactions.

## Supporting information

S1 Dataset Data from the study containing information about, patients, biopsies collected as well as regulatory T cell counts and *in situ* cytokine labeling. (XLSX)
